# Towards Machine-Readable (Meta) Data and the FAIR Value for Artificial Intelligence Exploration of COVID-19 and Cancer Research Data

**DOI:** 10.3389/fdata.2021.656553

**Published:** 2021-08-30

**Authors:** Maria Luiza. M. Campos, Eugênio Silva, Renato Cerceau, Sérgio Manuel Serra da Cruz, Fabricio A. B. Silva, Fábio. C. Gouveia, Rodrigo Jardim, Nelson Kotowski, Giseli Rabello Lopes, Alberto. M. R. Dávila

**Affiliations:** ^1^ Instituto de Computação, Universidade Federal do Rio de Janeiro, UFRJ, Rio de Janeiro, Brazil; ^2^ Unidade de Computação (Ucomp), Centro Universitario Estadual da Zona Oeste (UEZO), Rio de Janeiro, Brazil; ^3^ Instituto Nacional de Cardiologia, INC, Rio de Janeiro, Brazil; ^4^ Universidade do Estado do Rio de Janeiro, UERJ, Rio de Janeiro, Brazil; ^5^ Departamento de Ciências da Computação, Universidade Federal Rural do Rio de Janeiro, UFRRJ, Seropédica, Brazil; ^6^ PROCC, FIOCRUZ, Rio de Janeiro, Brazil; ^7^ Casa de Oswaldo Cruz, FIOCRUZ, Rio de Janeiro, Brazil; ^8^ Laboratório de Biologia Computacional e Sistemas, Instituto Oswaldo Cruz, FIOCRUZ, Rio de Janeiro, Brazil

**Keywords:** COVID-19, Findable, Accessible, Interoperable, and Reusable, cancer, metadata, artificial intelligence

## State of the Art

Even before COVID-19, the bioinformatics labs and life science industry were investing extensively in ecosystems of technological and analytical applications/appliances to store, curate, share, integrate, and analyze large amounts of data. With the pandemic coming at an accelerating pace, a series of global research actions are being implemented to strive against the virus and its effects and to create data-driven investigations to support more agile responses to future events^1^. Innovative solutions in COVID-19 research require more efficient and effective data management strategies and practices. Cancer research is an excellent example of the adoption of the FAIR (Findable, Accessible, Interoperable, and Reusable) data principles (Wilkinson et al., 2016) on precision oncology (Deist et al., 2020; Delgado and Llorente, 2020; Vesteghem et al., 2020) and major cancer data repositories, such as the NIH Cancer Research Data Commons, are gradually adhering to these principles.

In a broad sense, understanding the whole scenario of a worldwide virus outbreak such as the COVID-19 pandemic has been a great challenge. It involves a huge effort to put together facts and a large volume of data related to the dynamics of the real world, as well as past and current results of scientific research. At the same time, cancer researchers are pivoting portions of their investigations and contributing with their expertise and resources to scientific studies of the SARS-Cov-2. Their findings have been wide in scope, ranging from insights to researching how the virus enters cells to identify potential therapies (NCI Staff, 2021).

Today, researchers are running against the clock to face at least three barriers to run data-driven investigations. First, data specialists refer to the fact that approximately 80% of researchers’ time is spent finding, cleaning, and organizing data (Schrage, 2017; Tyagi, 2020). Second, the availability of research data declines quite rapidly as articles age (Miyakawa, 2020). Third, raw data supporting the scientific results or proper descriptions of data repositories (Vines et al., 2014) are lacking in many investigations and articles. These barriers may hinder innovation and scientific development because they increase the so-called “reproducibility crisis” of scientific experiments. Hence, to circumvent such shortcomings and optimize researchers’ efforts and time, some research organizations (like the Research Data Alliance^2^, World Data Systems^3^, GO FAIR^4^, etc.) are discussing how data initiatives can be properly incorporated into the life cycle of data-driven experiments, aiming to increase preservation, and sharing and reuse of data.

## COVID-19 and Lessons Learned From Cancer Research

The explosion of biomedical big data has considerably changed the landscape of cancer research. Researchers are used to dealing with complex biological problems and carrying out heterogeneous data-driven investigations (Schade et al., 2019; Bailey et al., 2020). It is a consensus that single research centers cannot produce enough data to fit prognostic and predictive models of sufficient accuracy. Hence, data integration in precision oncology is of great relevance (NCI Staff, 2021).

Nowadays, large-scale COVID-19 and precision oncology projects face several challenges (Budin-Ljøsne et al., 2014; Bertier et al., 2016). Some issues lie in the ways data are recorded, stored, and reused. In addition, various health-care systems are incompatible, making it difficult, expensive, and time-consuming to aggregate datasets from different sources due to the diversity of data involved and poor data management (Vesteghem et al., 2020).

Even before the coronavirus pandemic, various European cancer initiatives have emerged to tackle these issues by standardizing and facilitating data pipelines. Several groups are implementing the FAIR data principles, fostering the use of standards, common metadata models, and ontologies to increase the interoperability and reusability of data in oncology projects (Martínez-García et al., 2020; Zong et al., 2020).

Data stewardship is an essential driver of cancer research groups and clinical practice. Since 2016, the FAIR data principles have been resonating in scientific health communities. Enabling data to be FAIR is currently believed to strengthen data sharing, reduce duplicated efforts, make them more findable by machines, and harmonize data from heterogeneous unconnected data silos. These lessons learned by cancer researchers can minimize future health emergencies and humanitarian crises in all countries regarding COVID-19.

## The COVID-19 Case and FAIR Initiatives

The FAIR guiding principles (Wilkinson et al., 2016) were created to save researchers’ time and help maximize the impact of health data. The principles began in a few European academic institutions and have burgeoned to include endorsements by global organizations such as the Group of Seven (G7) intergovernmental organization, science funding agencies, and national governments. They are a fundamental enabler of digital transformation and data interoperability in data-driven computing applications. These principles aim to enhance the ability of machines to automatically find and use (meta)data (Heath and Bizer, 2011).

Several challenges are related to the discovery, access, and interoperability of data from different sources. Before the FAIR principles were proposed, a set of principles and technologies, known as Linked Data, used the Web infrastructure to enable data sharing and reuse on a massive scale (Bizer et al., 2009), creating the Web of Data. The Linked Data principles (Semantic) are a set of best practices for publishing structured data on the Web, including the following: (i) to use URIs (Uniform Resource Identifiers) as names for things; (ii) to use HTTP URIs so that people can look up those names; (iii) when someone looks up a URI, to provide useful information, using standards like RDF and SPARQL; and (iv) to include links to other URIs so that more resources can be discovered. It is also essential to highlight the need to use controlled vocabularies and ontologies as well as establishing interlinks for proper exploration of the Web of Data.

The FAIR principles are focused on research data and are not limited to specific technologies, but they can benefit from Linked Data technologies. FAIR also includes a stated license for access, not addressed by the open nature of Linked Data (Heath and Bizer, 2011). In this sense, the FAIR Data Point was inspired by the Linked Data platform but targets its development more explicitly related to the FAIR principles (Wilkinson et al., 2016). A FAIR Data Point stores information about the datasets, that is, metadata, both human and machine-readable.

The preparation of data to properly interoperate and be reused can be improved dramatically by implementing the FAIR data principles for scientific data management. Thus, many powerful analytical tools such as machine learning algorithms and artificial intelligence (AI) packages will automatically access the data from which they learn and extract new knowledge. Moreover, in previous stages, machine learning, AI, and data mining techniques can also be instrumental: (i) in the step of data preparation, for example, helping to transform nonstructured data for the publication as structured data, following the Linked Data principles; (ii) in other steps, as in discovering vocabularies and ontologies to annotate the (meta)data or in identifying new datasets for interlinkages.

Thus, the FAIR principles and associated infrastructure can undoubtedly contribute to creating a federated network of data distribution associated with different aspects of the COVID-19 pandemic as well as cancer research. The ideas proposed by the GO FAIR initiative received unprecedented attention. They were endorsed by research data communities that valued the contribution of the GO FAIR movement in putting a lot of emphasis on (meta)data publishing protocols, semantic support, and machine actionable elements.

At the beginning of 2020, the Virus Outbreak Data Network (VODAN)^5^ was conceived as an implementation network collaboratively developed to support the capture and use of data, following the FAIR data principles, not only during this pandemic but also on future infectious disease outbreaks. The network serves both human and machine exploration, fostering the reuse and reproducibility of scientific resources. The seed of the VODAN BR project^6^ started to implement one of the network data points, with pilot collaboration, collecting, and treating anonymized patients’ data from COVID-19 cases of two public hospitals, following the World Health Organization standard form.

The VODAN BR project started at the beginning of the Brazilian COVID-19 outbreak. The primary goals were to understand the value of FAIR data management through the appropriate education and training of several researchers and health staff, combined with the necessary cultural change motivation. After that, the technological infrastructure to support the FAIR data life cycle was developed and is still under improvement.

Through the so-called FAIRification process, both data and metadata become machine-processable, receive permanent identifiers, and are associated with vocabularies and ontologies to reduce ambiguity. The data licensing scheme may vary from allowing complete open access to more restricted access for research partners only. The metadata, on the other hand, are published in federated FAIR Data Points for open access, and, more importantly, as metadata for the machine (M4M)^7^ to be automatically processed or human consumed.

Our general goals are aligned with global efforts developed by the pharmaceutical industry, and other life sciences R and D such as biomedicine, environmental sciences, agriculture, and food production. For instance, there are several successful cases in the big pharma industry. AstraZeneca intensified the use of identifiers to find and access internal data. Roche, Bayer, and SciBite demonstrated the value of interoperability through linked (meta)data (Hasnain et al., 2018). Nevertheless, the major value of FAIR data to the VODAN BR project and any other organization is the larger reusability beyond the initial and primary purpose of the datasets (Wise et al., 2019).

The FAIRification process enables us to achieve more value from internal and external data over a greater period. The associated linked provenance and general metadata are expected to persist as a permanent scientific record, even when the original data have lost value and have been archived or eventually deleted as a part of the FAIR data management life cycle.

In the present pandemic scenario, actions that can amplify data sharing, and globally contribute to research development—with carefully defined levels of openness to protect sensitive data—are key for generating rapid and coordinated responses from science. Briefly, VODAN BR is one of these efforts, dealing with the challenges of establishing a distributed infrastructure to harvest COVID-19 semantic (meta)data. It starts by addressing patients’ data and is then expected to evolve by effectively supporting interoperability with many other distributed datasets using artificial intelligence, machine learning, and data science algorithms to assist health teams and public managers in making better data-driven decisions.

Last but not least, considering that data from omics sciences are produced at an unprecedented speed and volume, surpassed only by data produced by astronomers (Stephens et al., 2015), adopting the FAIR principles is undoubtedly critical to support genomic-based research and discoveries globally.

## Footnotes

1 Establishing a FAIR Biomedical Data Ecosystem: The Role of Generalist Repositories to Enhance Data Discoverability and Reuse Workshop. February 11, 2020–February 12, 2020–NIH Main Campus (https://datascience.cancer.gov/news-events/events/establishing-fair-biomedical-data-ecosystem-role-generalist-repositories-enhance).

2
https://www.rd-alliance.org/

3
https://www.worlddatasystem.org/

4
https://www.go-fair.org/

5
https://www.go-fair.org/implementation-networks/overview/vodan/

6
https://vodanbr.github.io/

7
https://www.go-fair.org/how-to-go-fair/metadata-for-machines/
